# Peculiarities of Neurostimulation by Intense Nanosecond Pulsed Electric Fields: How to Avoid Firing in Peripheral Nerve Fibers

**DOI:** 10.3390/ijms22137051

**Published:** 2021-06-30

**Authors:** Vitalii Kim, Emily Gudvangen, Oleg Kondratiev, Luis Redondo, Shu Xiao, Andrei G. Pakhomov

**Affiliations:** 1 Frank Reidy Research Center for Bioelectrics, Old Dominion University, Norfolk, VA 23508, USA; vkim@odu.edu (V.K.); egudvang@odu.edu (E.G.); SXiao@odu.edu (S.X.); 2 Energy Pulse Systems, 1600-546 Lisbon, Portugal; oleg.kondratiev@energypulsesystems.com; 3 Lisbon Engineering Superior Institute, GIAAPP/ISEL, 1959-007 Lisbon, Portugal; lmredondo@deea.isel.ipl.pt; 4 Department of Electrical and Computer Engineering, Old Dominion University, Norfolk, VA 23508, USA

**Keywords:** excitation, electropermeabilization, electrostimulation, nsPEF, nerve stimulation, electroporation, nanoporation

## Abstract

Intense pulsed electric fields (PEF) are a novel modality for the efficient and targeted ablation of tumors by electroporation. The major adverse side effects of PEF therapies are strong involuntary muscle contractions and pain. Nanosecond-range PEF (nsPEF) are less efficient at neurostimulation and can be employed to minimize such side effects. We quantified the impact of the electrode configuration, PEF strength (up to 20 kV/cm), repetition rate (up to 3 MHz), bi- and triphasic pulse shapes, and pulse duration (down to 10 ns) on eliciting compound action potentials (CAPs) in nerve fibers. The excitation thresholds for single unipolar but not bipolar stimuli followed the classic strength–duration dependence. The addition of the opposite polarity phase for nsPEF increased the excitation threshold, with symmetrical bipolar nsPEF being the least efficient. Stimulation by nsPEF bursts decreased the excitation threshold as a power function above a critical duty cycle of 0.1%. The threshold reduction was much weaker for symmetrical bipolar nsPEF. Supramaximal stimulation by high-rate nsPEF bursts elicited only a single CAP as long as the burst duration did not exceed the nerve refractory period. Such brief bursts of bipolar nsPEF could be the best choice to minimize neuromuscular stimulation in ablation therapies.

## 1. Introduction

Treatments with intense PEF are central for many existing and emerging medical applications, including tumor and tissue ablations [1,2,3,4,5,6,7,8,9,10,11,12,13,14,15]. Ablations by PEF rely primarily on breaking the plasma membrane barrier of the cells by electroporation, leading to irreversible changes in homeostasis and cell death. PEF ablation destroys the cellular component of tissues while sparing the extracellular matrix. Such selectivity facilitates the orderly repopulation of treated areas by healthy cells and functional recovery.

Although PEF ablations are considered minimally invasive, the treatment may cause severe pain, involuntary muscle contractions, and heart fibrillation [7,16,17,18]. These major side effects need to be mitigated by local or general anesthesia and muscle relaxants, as well as by synchronizing PEF with nonvulnerable phases of the heart cycle. These side effects can be better appreciated by comparing the induced transmembrane potentials (TMP) for neuromuscular excitation and for electroporation. For conventional electric stimuli of 0.1–1-ms durations, the TMP threshold for electroporation is at 200–500 mV [19,20,21,22]. Achieving irreversible electroporation and cell death requires even higher TMP and delivering tens or hundreds of pulses. However, neuromuscular excitation takes a single pulse that induces a TMP of 10–20 mV. With the stimulation TMP thresholds being an order of magnitude smaller than the TMP required for ablation, the stimulated tissue volume is often much larger than the ablation volume.

Shortening the electric pulses in the sub-microsecond range reduces their efficacy to stimulate neurons and cardiomyocytes, and stimulation thresholds for nanosecond PEF (nsPEF) are often at or above the electroporation thresholds [23,24,25,26,27]. These and other data suggested that nsPEF may be a preferred modality for ablation with minimal stimulatory side effects [23,28]. Furthermore, changing the electric field direction, e.g., by applying bipolar pulses, inhibits stimulation with nsPEF [29]. This feature, called “bipolar cancellation”, is unique for nsPEF, including short microsecond range pulses, and disappears when the pulse duration is further increased. However, bipolar cancellation reduces the electroporation efficiency as well [30,31,32,33], and it is not known whether or under what conditions electrostimulation is inhibited stronger than electroporation.

The reduction of neuromuscular stimulation by applying shorter and bipolar pulses has been analyzed by numerical and analytical models [34,35,36,37,38,39,40], as well as in animal and in vitro experiments [28,41,42]. A number of studies have focused specifically on high-frequency irreversible electroporation (H-FIRE), which stands for a complex protocol of delivering bursts of symmetrical bipolar stimuli with 0.5–2-µs phase durations [34,35,36,43]. Both model computations and animal experiments have demonstrated successful tumor ablations by H-FIRE protocols while the neuromuscular stimulation was markedly reduced.

Although the computational models have demonstrated a lower efficacy of nsPEF for stimulation, they have not been validated for the nanosecond range by experimental data, which remain scarce or nonexistent. Known nsPEF effects such as the lasting inhibition of voltage-gated Na^+^ and Ca^2+^ channels [44,45], nanoelectroporation [46,47,48], TMP sensitivity of the nanoporated membrane [46,49], a minimum depolarization time for voltage-gated Na^+^ channels to open [29], a bipolar cancellation [50,51], and MHz compression effects [26,52] have not been understood well enough to be quantitatively incorporated into excitation models. For example, the electroporation of neurons and cardiomyocytes by nsPEF intensities lower than needed for excitation [23,24,25] has not been predicted by the models. The excitation of peripheral nerve fibers by nsPEF as short as 12 ns [53] and even 1 ns [42] without any concurrent membrane damage was a notable exception, possibly explained by a much larger membrane charging time constant compared to cells [23].

Peripheral nerve fibers are one of the best-studied excitable systems, but most model predictions and experimental data do not reach the nanosecond range and do not consider complex waveforms. The only experimental study of the strength–duration relationship down to 1 ns was performed in a frog gastrocnemius muscle, where the recorded muscle twitches were probably a result of the excitation of nerve terminals within the muscle [42]. The goal of our study was to validate the strength–duration results in a peripheral nerve submerged in a conductive medium and to include bipolar (bi- and triphasic) and high-repetition rate nsPEF stimuli. We chose to use frog nerves due to their resilience to in vitro conditions, with the understanding that the quantitative data may differ from mammalian nerves. It is more important that the fundamental mechanisms of nerve excitation are highly conserved in vertebrate animals; hence, the rules on how the nerves respond to different stimuli should be universal across the different species. We explored the “worst-case scenario” for ablation, i.e., looked at the responses of the most excitable nerve fibers and applied stimuli in a way that caused excitation at the lowest electric field threshold.

## 2. Results

### 2.1. Nerve Excitation by Nano- to Millisecond Pulses

**Figure 1:**
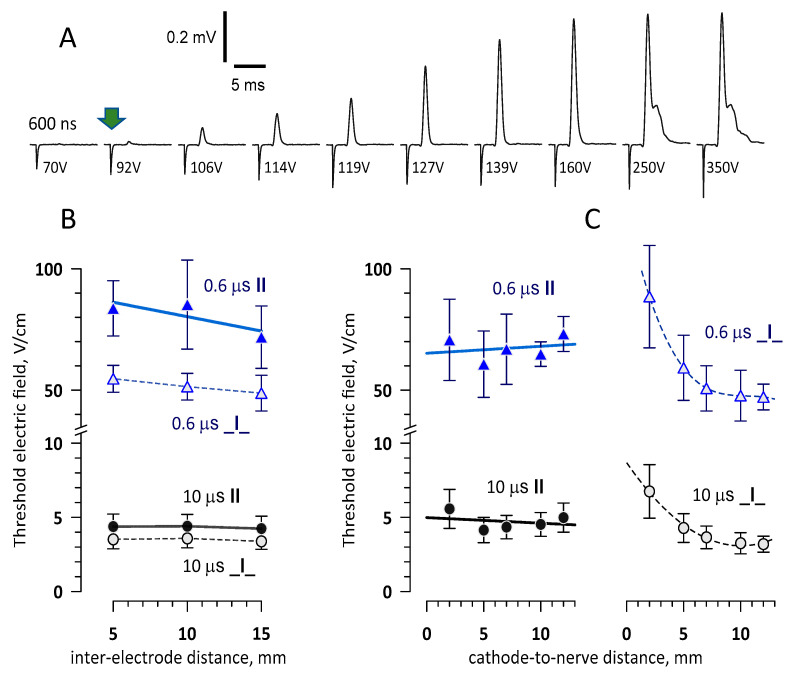
Sample traces of the compound action potentials (CAPs) evoked in a frog sciatic nerve (**A**) and the effect of the stimulating electrode placement on the excitation threshold (**B**,**C**). (**A**) The nerve was submerged in the physiological solution and stimulated with a bipolar electrode by a single 600-ns pulse at the indicated voltage. The arrow identifies the stimulation threshold when the CAP amplitude reached about 20 µV. (**B)** The effect of the inter-electrode distance on the electric field threshold for 0.6-µs and 10-µs pulses. The electrodes were placed 7 mm from the nerve either parallel (**II**) or perpendicular (**_I_**) to it. The cathode was closer to the recording electrodes (**II**) or closer to the nerve (**_I_**). (**C)** The effect of the cathode-to-nerve distance for the **II** and **_I_** placements of the electrodes. The inter-electrode distance was 15 mm. Mean values ± SE, *n* = 5.

As described below in Methods, the stimulated portion of the nerve was submerged in the physiological solution, and the stimuli were delivered with a bipolar electrode (a pair of blunt needles). In most experiments, 0.5-mm diameter needles were immersed in the solution in a plane orthogonal to the nerve, either touching it or at a distance. The stimulation was considered to be at the threshold when it evoked a minimally discernable compound action potential (CAP), with a peak amplitude of about 20 mV (Figure 1A). Trapezoidal electric pulses of all the tested pulse durations (10 ns^–1^ ms) evoked CAPs repeatedly and consistently. The threshold voltage expectedly increased for shorter pulses and with the distance from the nerve. The respective threshold electric field at the nerve location closest to the electrodes stayed the same or even decreased with the distance (see Section 2.2). Stimulation at about two-fold the threshold voltage excited all fast fibers and increased the CAP to a maximum of 0.5–2 mV (Figure 1A).

Consistent with the previous reports [26,29,53], each nerve preparation withstood thousands of excitation cycles with little or no signs of rundown for 5–10 h. Nerve impairment by the electrostimulation (manifesting as a quick CAP decline) was rare and was only observed after repeated applications of supramaximal stimuli with the electrodes in contact with the nerve.

### 2.2. Placement of the Stimulating Electrodes and PEF Thresholds for Nerve Excitation

This set of experiments was aimed at identifying the stimulation geometry to elicit CAPs at the lowest PEF intensity. In tumor ablation treatments, such a geometry would correspond to the “worst-case scenario”, i.e., nerve excitation farthest from the ablation target. We varied (a) the distance between the stimulating electrodes (5–15 mm), (b) their orientation parallel or perpendicular to the nerve, (c) the nerve placement within or outside the gap between the electrodes, (d) the distance from the electrodes to the nerve (2–12 mm), and (e) the polarity (cathode or anode closer to the nerve for the perpendicular orientation, or cathode or anode closer to the recording electrodes for the parallel orientation). These conditions were tried in five or more nerve preparations with at least two pulse durations (600 ns and 10 µs) of unipolar PEF. The maximum electric field strength reached at the nerve location was calculated for all the different electrode placements by numerical simulations (see Methods).

The lowest PEF thresholds were measured with electrodes placed (1) parallel to the nerve, with the cathode closer to the CAP recording site, and (2) perpendicular to the nerve, when the nerve was outside of the inter-electrode gap and closer to cathode (Figure 1B,C). For both electrode placements and for both pulse durations, the PEF thresholds did not significantly change when the inter-electrode distance was varied from 5 to 15 mm (Figure 1B). Increasing the distance between the electrodes and the nerve did not affect the PEF threshold for the electrodes placed parallel to the nerve but decreased it for the perpendicular configuration. The threshold fell sharply from 2 mm to 5–7 mm and then stayed practically constant (Figure 1C).

The perpendicular configuration with the cathode placed 5 mm from the nerve was selected to determine the lowest PEF thresholds for the strength–duration curve (Section 2.3). Placing the cathode further away at 7 or 10 mm could provide a slight additional PEF threshold reduction, but exciting the nerve from this distance would require prohibitively high pulse voltages, especially for shorter pulses.

### 2.3. The Strength–Duration (S–D) Curve from 10 ns to 1 ms for Uni- and Bipolar PEF Stimuli

**Figure 2:**
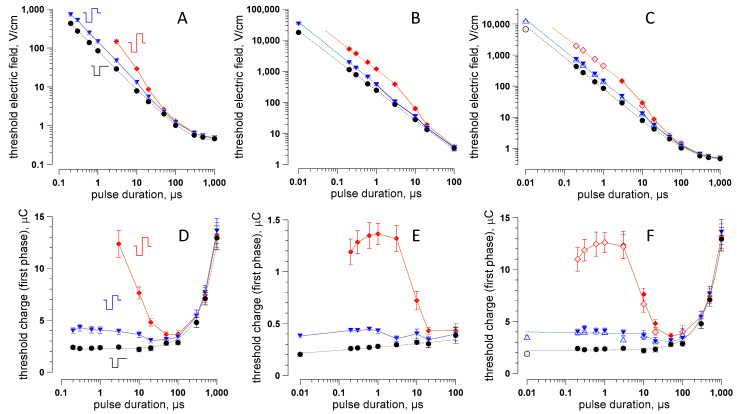
Strength–duration curves (**A**–**C**) and the respective electric charge thresholds (**D**–**F**) for unipolar cathodic pulses and bipolar pulses with the 2nd (anodic) phase 50% or 100% of the 1st (cathodic) phase. Electrodes were placed perpendicular to the nerve at a 5-mm distance to the cathode (**A**,**D**) or with the cathode touching the nerve (**B**,**E**). Panels (**C**,**F**) combine the thresholds measured at 5 mm (solid symbols) and those extrapolated from 0 mm to 5 mm (open symbols; see text for details). Dashed lines are data fits using the Lapicque formulation (**A**,**D**) or power function (**B**–**F**); solid lines just connect the mean values. Mean ± SE, *n* = 8–14 for most datapoints. Error bars may be not visible when they are smaller than the central symbol.

With stimulating electrodes placed perpendicular to the nerve, the cathode 5 mm and anode further 15 mm away, we were able to evoke CAPs by unipolar stimuli for 200-ns–1-ms durations (Figure 2A). For the 10-ns stimuli, the maximum voltage we could generate (~20 kV) was not sufficient to evoke CAPs from a 5-mm distance. The S–D curve for unipolar cathodic stimuli (200 ns–1 ms) had the classic shape and followed the Lapicque formulation [39,54] for the electric field strength:

E_T_ = E_0_/(1 − exp(−*t*/τ_e_)), (1)

where E_T_ is the threshold PEF strength at the pulse duration *t*, E_0_ is the minimum electric field threshold for the longest pulses (the rheobase), and τ_e_ is the empirical time constant (170 µs for unipolar PEF in Figure 2A). This S–D curve closely matched the one for muscle stimulation [42], with the nerve E_T_ values 10–50% lower for most PEF durations.

The addition of the opposite polarity (anodic) second phase at either 50% or 100% of the first phase amplitude did not affect the threshold for PEF longer than 50 µs (Figure 2A). Indeed, the CAP was elicited during the first phase and already traveling away before the second phase started. At a 10–50-µs phase duration, the bipolar PEF thresholds gradually became higher than the unipolar ones. Since the CAP starts only at 10 µs or later after the stimulus onset [29], it was generated during the second phase or after it, during the membrane repolarization after the anodic phase. In the first scenario, the “early” portion of the second phase reduced the depolarizing effect of the first phase, while the remaining “late” portion played no role. In the second scenario, the entire second phase reduced the depolarization and increased the electric field threshold.

We were able to evoke CAPs by PEF down to 200 ns with 50% bipolar pulses but only down to 3 µs with 100% bipolar pulses. For still shorter pulses, the excitation from a 5-mm distance was beyond the voltage output limits of our pulse generators. Hence, we had to bring the cathode into contact with the nerve to measure the thresholds from 10 ns to 100 µs (Figure 2B). The inter-electrode distance was kept unchanged at 15 mm. For 100% bipolar PEF with a 10-ns phase duration, stimulation at 20 kV (40 kV peak-to-peak) was still not enough to elicit CAPs, even with the cathode touching the nerve, so the threshold data were limited to 200 ns.

The ratio of the thresholds measured at 5 and 0 mm from the nerve with unipolar pulses was the same for different pulse durations (data not shown). The average value of this ratio was taken as a coefficient to extrapolate threshold measurements for bipolar pulses from 0 to 5 mm. The extrapolated and measured thresholds (open and filled symbols, respectively, in Figure 2C) matched well for all the datapoints where this comparison was possible. With this extrapolation still taken with reasonable caution, the S–D curves in Figure 2C represent our best estimate for the lowest PEF thresholds for nerve excitation from 10 ns to 1 ms by unipolar and 50% and 100% bipolar pulses.

For all the studied pulse types, the S–D curves in the nanosecond range were linear in the double-logarithmic coordinates and parallel to each other. Fitting the thresholds for nsPEF by a power function (dashed lines in Figure 2C):

E_T_ = ***a*** × *t***^b^** (2)

yielded the same slope coefficient **b** of −1.01, −1.01, and −0.97 for unipolar, 50% bipolar, and 100% bipolar nsPEF, respectively. In other words, the nsPEF thresholds were inversely proportional to the pulse (or the first phase) duration, E_T_ = ***a***/*t*, irrespective of the presence or relative amplitude of the second phase. The multiplier ***a*** equaled 82, 135, and 425 for the unipolar, 50%, and 100% bipolar nsPEF, respectively. In other words, the threshold ratio for the three types of nsPEF was 1:1.6:5.2 regardless of the pulse duration. The addition of the second phase always made nsPEF less efficient during the nerve stimulation. The reduction of the stimulation efficiency was determined by the second phase amplitude uniformly for different pulse durations in the nsPEF range.

### 2.4. The Role of the Electric Charge in Nerve Stimulation by nsPEF

The multiplier ***a*** = E_T_ × *t* is, by definition, a value linearly proportional to the electric charge. Indeed, the S–D curves replotted as the 1st phase charge at the threshold are flat for pulse durations under 10 µs (Figure 2D–F). This result is consistent with the model predictions for unipolar pulses shorter than 10 µs [39]. Our data validated this prediction and extended it to short 50% and 100% bipolar stimuli. For 100% bipolar pulses, the remarkable transition from a steep threshold rise (50–10 µs) to a plateau (<3 µs) matched the critical phase duration where the transition from the cathodic (by the first phase) to the anodic stimulation (by the second phase only) was expected [29]. However, the plateauing of this threshold contrasted its increase for still shorter pulses, which was reported earlier [29] and also predicted by modeling [43]. Due to this difference, we performed an additional series of experiments aimed specifically at the verification and replication of the data in question. This replication series fully confirmed the initial observations. At this point, we do not have a clear explanation of this result and can only speculate that it was related to the stimulation in the volume of a conductive saline and the asymmetric placing of the electrodes perpendicular to the nerve (as opposed to two electrodes locally touching the nerve coated with an insulation material [29]).

### 2.5. The Interplay of Phase Amplitudes and Their Ratio in Nerve Excitation by Bipolar nsPEF

Figure 2D–F shows that the charge threshold of the first phase does not depend on the pulse width in the nanosecond range but changes with the addition of the second phase. Our next experiments explored how the ratio of the two phases and the differences in their voltage affect the excitation threshold by bipolar nsPEF.

Stimulating electrodes were placed perpendicular to the nerve, with the cathode touching it and the anode 15 mm away, and were energized from an EPULSUS nsPEF stimulator (see Methods). This device utilizes two separate eight-stage Marx generators to deliver cathodic and anodic phases of a bipolar pulse. Each stage of both Marx generators is charged to the same voltage from a single power supply, but the output voltage is determined by the number of stages engaged to produce each phase. For example, we could engage four and three stages of the two generators to maintain a constant 4/3 (75%) ratio of the first phase to the second one, irrespective of the charging voltage from the power supply. Likewise, we could engage four and four stages to maintain a symmetrical bipolar pulse or four and six stages to always keep the second phase 50% larger than the first one, etc.

Engaging different numbers of stages enabled a coarse control of the charge ratio of the first and second phases. For a finer control, we kept the first phase duration constant at 600 ns and varied the second phase duration from 600 to 750 ns. The increase of either the phase duration or voltage had the same impact on the electric charge. To facilitate the data analysis, we adjusted the second phase voltage to a charge-equivalent voltage of a 600-ns phase (V_**600**_):

V_**600**_ = V***_D_*** × ***D***/600 (3)

where V***_D_*** is the voltage at a phase duration ***D*** in nanoseconds.

The experiments started with determining the threshold for a unipolar 600-ns cathodic pulse. Next, the second (anodic) phase was added and tuned to a selected charge ratio to the first phase (from 0% to 200%). Next, the power supply voltage was gradually increased, leading to a concurrent increase of both phases while keeping their charge ratio constant. Once a new excitation threshold was reached and recorded, the experiment was repeated with a different ratio of the two phases and so forth.

**Figure 3:**
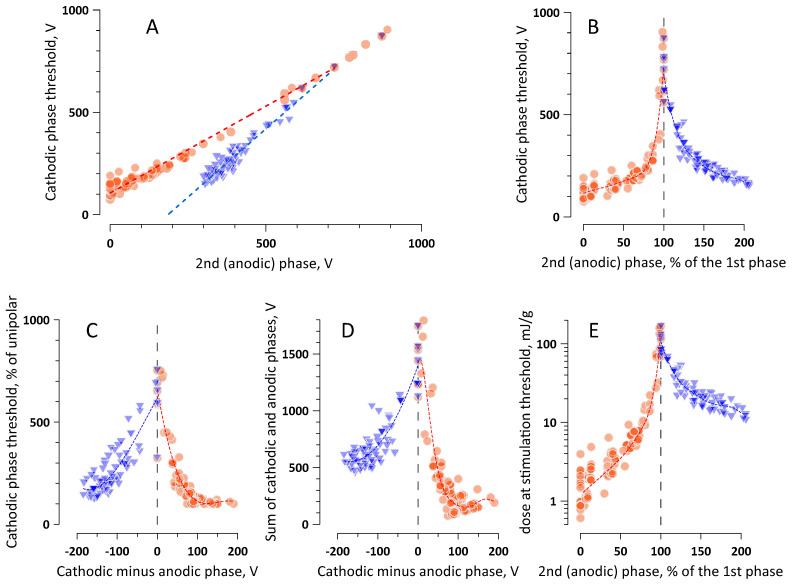
Nerve excitation by (600 + 600)-ns bipolar stimuli with different balance of the cathodic (1st) and anodic (2nd) phases, see text for details) Orange circles are the datapoints for the cathodic stimulation (larger 1st phase), and blue triangles are for the anodic stimulation (larger 2nd phase); the datapoints for symmetrical bipolar pulses were included in both groups. (**A**–**D)** The impact of the 2nd phase amplitude (**A**,**B**) or the difference between two phases (**C**,**D**) on the cathodic phase threshold voltage (**A**–**C**) or the sum of voltages of both phases at the excitation threshold (**D**). (**E**) The absorbed dose at the excitation threshold for pulses with different ratios of the 1st and 2nd phases. The data are from 19 experiments in 4 nerve preparations. The dashed lines are linear fits in (**A**) and polynomial fits in the other panels.

The threshold data were plotted in different ways (Figure 3A–D), all showing that symmetrical bipolar pulses require the highest amplitude of both phases to reach excitation. (For bipolar pulses with different durations of two phases, “symmetrical” would mean that the phases have the same charge, i.e., the same area under the voltage trace.) Asymmetrical pulses always evoked CAPs at smaller amplitudes of both phases by either cathodic stimulation (a larger first phase) or anodic stimulation (a larger second phase), with the latter one being expectedly less efficient [39]. The threshold voltage of the first phase decreased as the pulses became “less symmetrical”, reaching one minimum when the second phase dropped to zero and reaching the other (larger) minimum when the second phase was made two-fold larger than the first one.

A closer look at the data, especially in Figure 3C,D, suggests that bipolar pulses with the first phase just slightly larger than the second phase may be even less efficient at stimulation than the perfectly symmetrical pulses. This observation is in line with the predictions of the capacitor model of nerve excitation by bipolar nsPEF [29]. However, the tiny difference of 8–15 V between two phases as large as 1500–1900 V each (i.e., <1%) is difficult to prove, as this value is close to the accuracy limits of voltage measurements. The capacitor model also predicted that, at a certain perfectly chosen difference between the two phases, the excitation threshold becomes infinitely high, but it may take a single isolated nerve fiber instead of a nerve trunk to test this prediction experimentally.

The steep rise in the threshold amplitude of both phases for charge-balanced symmetrical nsPEF is emphasized by a 100-fold increase in the absorbed dose compared to a unipolar cathodic pulse and a 10-fold increase compared to a unipolar anodic pulse (Figure 3E; note the log scale). The absorbed dose was calculated as described earlier [55], using the actual values of the amplitude and duration of each phase (without normalization to a 600-ns pulse). Even the highest required dose corresponded to the adiabatic heating of less than 0.2 °C. However, heating may become damaging and should be considered with burst stimulation by symmetrical nsPEF at MHz repetition rates.

### 2.6. The Time-Average Voltage across Multiple Pulse Phases (=Net Charge) Predicts the Efficacy of Cathodic and Anodic Stimulation by Complex nsPEF Shapes

**Figure 4:**
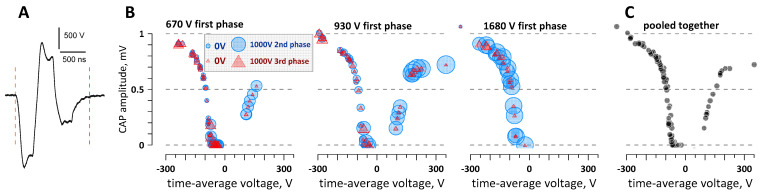
The time-average voltage measured across a triphasic stimulation pulse determines the amplitude of the evoked compound action potential (CAP). The data are from one representative experiment using a triphasic pulse (**A**) with adjustable amplitudes of each phase. The stimulating voltage was automatically averaged across the interval between the vertical dashed lines and used for abscissa values in (**B**,**C**). The vertical scale in (**B**,**C**) is the elicited CAP amplitude. The three panels in (**B**) are for three discrete amplitude values of the 1st (cathodic) phase of the pulse, as shown in the legends. The amplitudes of the 2nd and the 3rd phases are coded by the symbol size, with the legend and symbol size scaling shown in the left panel. (**C**) All the datapoints pooled together and plotted irrespective of the individual phase amplitudes.

Figure 4 shows a representative experiment (out of four) where the net electric charge from two or three PEF phases crossed the zero line and evoked CAPs by either cathodic or anodic stimulation. The stimulating electrodes were placed parallel to the nerve at a 3-mm distance, with the cathode closer to the CAP recording site. Each phase was trapezoidal, with an initial higher peak followed by a gradual descend and a plateau (Figure 4A). The amplitude of each phase could be adjusted independently and was measured at the eyeballed middle point between the peak and the plateau. The duration of each phase at 50% height was around 300 ns and varied with the phase amplitude. With such a complex shape, it was difficult to measure the electric charge delivered by each phase. Instead, we used the integration function of the oscilloscope to measure the average voltage over a constant time interval slightly longer than the entire triphasic pulse (between the two dashed vertical lines in Figure 4A). This time-average voltage was proportional to the net electric charge, regardless of the variations of the amplitudes and shapes of the individual phases.

The first phase (cathodic) had three fixed amplitude settings (1680, 930, and 670 V) and was followed by an anodic phase that was varied from 0 to 1300 V and then by a second cathodic phase of 0–1090 V. Figure 4B shows the dependence of the evoked CAPs on the amplitudes of the three phases of the stimulation pulse. It is split into three panels, one panel for each of the three tested amplitudes of the first phase. The amplitudes of the second and third phases are presented by “bubble plots”, with the size of the semi-transparent “bubble” proportional to the phase amplitude (size calibration is shown in the left panel). Figure 4C shows all the datapoints brought together in one plot, without distinction between the individual phase amplitudes.

The plots in Figure 4B,C show that it was in fact the time-average voltage that determined the CAP amplitude. The stimuli were evoked by either cathodic stimulation (higher first and third phases; negative time-average voltage) or anodic stimulation (higher second phase; positive time-average voltage). Within the studied limits, the average voltage determined the electric charge and CAP amplitude regardless of the exact pulse shape or the ratio of amplitudes of its individual phases. Based on the previous set of experiments (Figure 3), one can anticipate that CAPs could be evoked at near-zero time-average voltage values as well but only at phase amplitudes higher than we could reach in this experiment.

### 2.7. The Reduction of the Excitation Threshold by High-Rate Bursts of Uni- and Bipolar nsPEF

The temporal summation of the nsPEF stimuli was demonstrated recently for 5- and 100-pulse bursts of 340-ns unipolar stimuli [26]. The excitation threshold stayed at the same level as for a single pulse until a critical duty cycle of about 0.1% was exceeded. Afterwards, the threshold decreased rapidly as a power function of the duty cycle and then gradually approached the threshold for a single “long” pulse (i.e., a pulse that would be formed by the nsPEF burst at the 100% duty cycle).

**Figure 5:**
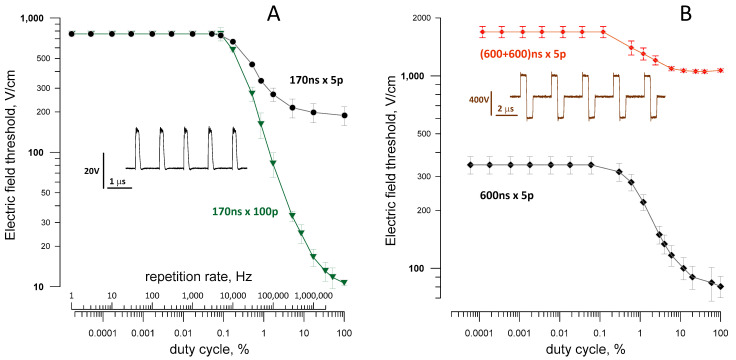
Temporal summation reduces the excitation thresholds for high-rate nsPEF bursts. (**A**) The effect of the duty cycle and pulse repetition rate on the excitation thresholds for bursts of 5 and 100 unipolar 170-ns pulses (170 ns × 5p and 170 ns × 100p). The inset is a sample waveform for a 5-pulse burst at 1 MHz. (**B**) Different efficacy of the threshold reduction for bursts of unipolar and symmetrical bipolar pulses (600 ns × 5p and (600 + 600) ns × 5p). The inset is a sample waveform for a 5-pulse burst of bipolar pulses at 0.33 MHz. Mean ± SE, *n* = 3–7.

Here, we validated the previous observations for bursts of shorter (170 ns) and longer (600 ns) unipolar pulses (Figure 5). We used different pulse generators (BNC for Figure 4A and EPULSUS for Figure 4B; see Methods) and also positioned the electrodes differently from the previous study (perpendicular to the nerve, with the cathode touching it and the anode 10 mm away). Notwithstanding these differences, the reduction of the threshold with increasing the pulse repetition rate followed the same pattern, with a critical duty cycle value at about 0.1%.

This duty cycle was also the same for symmetrical bipolar nsPEF, but the reduction of the threshold at high repetition rates was far weaker than with a similar burst of unipolar pulses (Figure 5B). Specifically, for a burst of five 600-ns pulses, the temporal summation reduced the electric field threshold 4.3 times for unipolar pulses but only 1.6 times for bipolar pulses (Figure 5B). The smaller threshold reduction may be particularly useful for the reduction of neuromuscular effects when performing tissue ablations with high-rate bursts of bipolar nsPEF.

**Figure 6:**
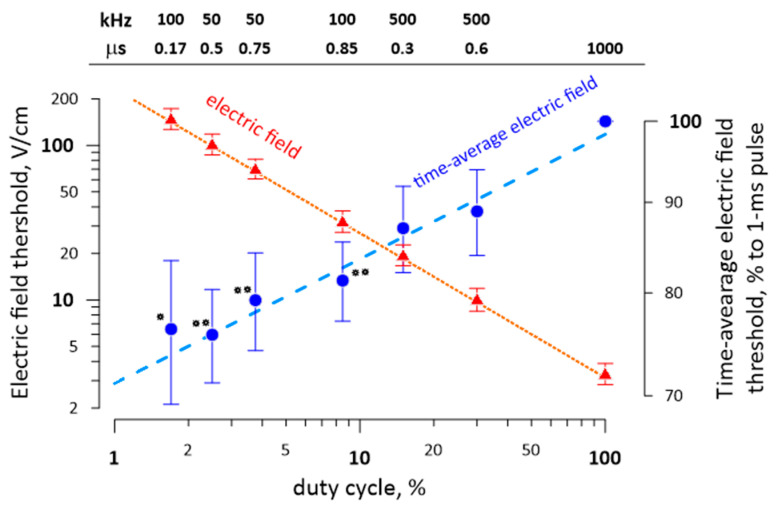
The electric field threshold for diverse 1-ms nsPEF bursts is determined by the duty cycle irrespective of the specific pulse duration or repetition rate (shown above each datapoint in µs and kHz, respectively). The “1000 µs” label is for a single 1-ms pulse. The blue symbols and the right vertical scale are for the electric field threshold averaged over the burst duration (1 ms) and normalized to the threshold for a single 1-ms pulse in the same nerve. Mean ± SE, *n* = 5. The time-average electric field threshold is significantly different from the threshold for 1-ms pulses (* *p* < 0.05 and ** *p* < 0.01, one-sample *t*-test). See the text for more details.

The role of the duty cycle as a critical parameter that determines the electric field threshold for different pulse durations and repetition rates was demonstrated in a separate experiment illustrated in Figure 6. We compared thresholds for 1-ms pulse bursts composed of different numbers of nsPEF, with varied durations and delivery rates. The electric field threshold fell from 200 V/cm to 2 V/cm as the duty cycle increased from 1% to 100%; the decline followed a power function irrespectively of the specific pulse duration or repetition rate within the burst.

We also measured a modest 20–30% reduction of the time-average electric field threshold when the duty cycle was reduced from 100% to 2% to 3% (Figure 6). Although this unexpected result was statistically significant (*p* < 0.01), it should be taken with caution. This change is just a fraction of a percent of the concurrent 100-fold change in the peak electric field and could potentially result from a less than perfect linearity of the measuring equipment and voltage probes.

### 2.8. Excitation by nsPEF Bursts during the Refractory Period

**Figure 7:**
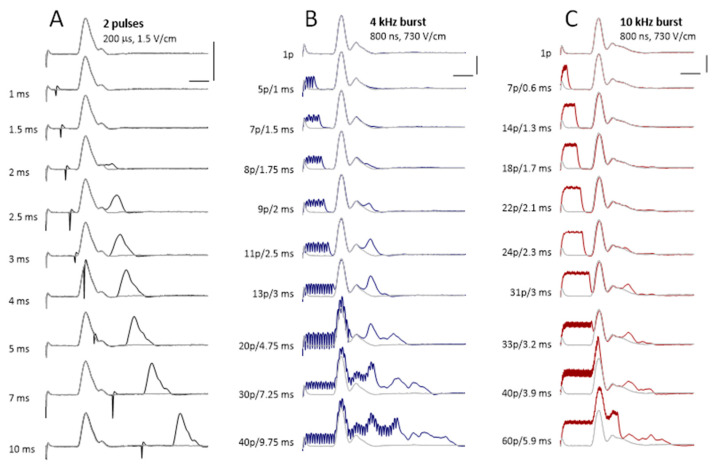
Sample traces of the compound action potentials evoked by paired pulses (**A**) and by 800-ns, 730-V/cm unipolar pulse bursts at 4 kHz (**B**) and 10 kHz (**C**). The interval between pulses (**A**) or the number of pulses per burst and the respective burst duration (**B**,**C**) were varied and are shown in the legends next to each trace. For ease of comparison, the top trace (CAP evoked by a single pulse) was copied behind all other traces as a thinner light-gray line. Calibration bars are 0.5 mV and 2 ms for all panels.

Nerve fibers become completely and then partially unexcitable immediately after generating an action potential (absolute and relative refractory periods). In a peripheral nerve, these periods are routinely measured by applying paired stimuli at different intervals, such as in Figure 7A, and comparing the amplitudes of CAPs evoked by the first and second stimuli. In the illustrated experiment, the absolute and relative refractoriness lasted less than 2 ms and about 10 ms after the first stimulus, respectively. If the refractory periods hold true for high-frequency nsPEF bursts, one would expect only a single nerve excitation from the first pulse in the burst, which could be an effective way to reduce the neuromuscular effects of ablation treatments.

We compared the refractory periods in nerves stimulated in a classic manner by paired pulses (Figure 7A) and by applying 4- and 10-kHz bursts of 800-ns unipolar pulses of supramaximal stimulation amplitudes (Figure 7B,C). The bursts included different numbers of pulses and, consequently, lasted different time.

With 4-kHz bursts, we could apply seven to eight pulses while still eliciting a single CAP. The duration of such bursts matched the absolute refractory period of less than 2 ms measured with paired pulses. Longer bursts evoked CAPs which appeared as several individual peaks (Figure 7B). The area under these additional peaks was proportional to the number of nerve fibers firing the second action potential after the refractory period. For a burst duration matching the inter-pulse interval for paired pulses, the number of fibers that recovered from the refractory period was approximately the same.

With 10-kHz bursts, the absolute refractoriness ended later, between 2.1 and 2.3 ms, and a burst of as many as 22 pulses produced no additional excitation compared to a single pulse (Figure 7C). Furthermore, bursts of up to 40 pulses (3.9 ms) excited much fewer nerve fibers than paired pulses applied at the same interval.

## 3. Discussion

We revealed some new and unexpected features of neurostimulation by uni- and bipolar nsPEF and quantified the nerve excitation for different pulse durations, pulse bursts, and phase ratios for bi- and triphasic pulses. In contrast to most experiments with isolated nerves where stimulating electrodes are placed directly on the nerve and are electrically insulated from each other, we delivered electric pulses into a volume of a conductive solution, with one or both electrodes placed at a distance from the nerve. The electrodes in the conductive medium produced a substantially different pattern of de- and hyperpolarization along the nerve fibers, which was a likely cause of some observations being different from previous reports [29]. Our settings provided a closer approximation of real-life conditions, when nerves are stimulated away from electrodes that target the ablation area. Whenever appropriate, we used the local electric field as a predictive parameter for nerve excitation, which also facilitates the utilization of the findings for in vivo conditions: a numerical simulation of the electric field in the tissues around the ablation target will immediately show where the nerve excitation is expected.

A number of our experimental findings with nsPEF stimulation are consistent with the predictions made by modeling the action potentials in peripheral nerves. These include increasing of the electric field threshold with pulse shortening down to 10 ns, higher thresholds for symmetrical bipolar nsPEF, a plateau of the threshold charge for unipolar nsPEF, and the importance of the net charge rather than the exact pulse shape [29,39,40,42,43]. However, some other features, like a reduction of the electric field threshold inversely proportionally to the pulse duration in the same fashion for unipolar, 50% bipolar, and 100% bipolar nsPEF, were not anticipated. Instead, we expected the strength–duration curve for 100% bipolar pulses to climb faster than for unipolar nsPEF when the stimulus duration decreases. Likewise, the experimentally measured dependence of the excitation threshold on the ratio (or voltage difference) between the two phases of a bipolar pulse did not confirm the expectations that a bipolar pulse with a somewhat smaller second phase will be entirely inefficient during nerve excitation [29]. It was not just the electric charge but a complex interplay of the charge and amplitude of each phase that determined the threshold for asymmetrical bipolar stimuli. The reduction of the excitation threshold and the key role of the duty cycle in high-rate burst stimulation were consistent with prior observations and modeling [26,52]. However, an incomparably weaker temporal summation and threshold reduction for symmetrical bipolar nsPEF has not been reported.

The mechanistic analysis of these phenomena and of the resulting quantitative differences in stimulation efficiency is beyond the scope of this work, which was aimed at defining the pathways to reduce neuromuscular stimulation as a side effect of electroablation. We looked for the electric pulse parameters and delivery protocols with minimal efficiency to evoke action potentials in a peripheral nerve. We found that the electric field threshold for nerve stimulation is increased by using nanosecond-range pulses and by adding a second phase to nsPEF but not to conventional “long” pulses. Symmetrical bipolar stimuli required a sharply higher electric field and energy to cause excitation than unipolar and asymmetrical bipolar nsPEF of the same duration. We also found that applying pulse bursts does not reduce the electric field threshold up to a critical duty cycle limit of 0.1%. While this critical duty cycle is the same for symmetrical bipolar nsPEF, the reduction of the threshold due to the temporal summation of individual pulses in the burst is not nearly as strong as with unipolar nsPEF. Stimulation by brief high-rate nsPEF bursts above the excitation threshold elicits only a single action potential by the first pulse, whereas the next pulses become entirely or partially inefficient when they fall within the absolute and relative refractory periods, respectively.

In the first approximation, tissue ablation by symmetrical bipolar nsPEF with the minimal phase duration, applied in brief bursts within the refractory period but not exceeding the critical duty cycle of 0.1% may be ideal to minimize neuromuscular effects. However, the same manipulations of the stimulation protocol (except for taking advantage of the refractory period) may reduce the ablation efficiency as well. For example, electroporation thresholds also increase for shorter pulses, but the numerical models suggested that this increase is less steep than for nerve excitation, and the border of excitation will be closer to the ablation border for shorter pulses [35]. Bipolar nsPEF are also less efficient at electroporation and cell killing than unipolar pulses, but, in contrast to the stimulation, the electroporation efficiency is minimal with the second phase at 50–75% of the first phase [31]. The maximum suppression of the electroporation is typically up to 3-fold [30,31,51,56], while the excitation thresholds are five to six times higher for symmetrical bipolar nsPEF than for unipolar ones (Figure 2 and Figure 3). A more detailed comparison of the impact of pulse parameters and protocols on ablation and stimulation will be reported in the next paper focused on the electric field thresholds for electroporation and cell killing. The differences in the cell damage and excitation thresholds will be analyzed and exploited to design ablation protocols with minimal neuromuscular effects.

## 4. Materials and Methods

### 4.1. Nerve Preparation

The animal procedures were the same as described previously [26,29] and were approved by the Old Dominion University Institutional Animal Care and Use Committee. Adult bullfrogs (*Rana catesbiana*) were euthanized by pithing the brain and the spinal cord. The sciatic nerves from both legs (*n. ischiadicus* + *n. peroneus*) were isolated and ligated at the proximal and distal ends. The nerves were submerged in a chilled physiological solution containing (mM): 140 NaCl, 5.4 KCl, 1.5 MgCl_2_, 2 CaCl_2_, 10 glucose, and 10 HEPES (pH 7.3, 290–300 mOsm/kg, 1.6 S/m). Usually, one of the nerves was used in the experiments immediately, while the other one was stored in the refrigerator for the next day.

### 4.2. Nerve Stimulation and CAP Recording

An isolated nerve was expanded in a custom stimulation/recording chamber made of a 135-mm Petri dish with an array of parallel metal pins mounted on its side [26]. The dish was filled with the physiological solution to a depth of 8 mm. Stimuli were applied to the distal end of the nerve submerged in the physiological solution, and CAPs were recorded from its proximal end pulled out of the solution and placed over the pins. The length of the nerve outside the solution was covered with a Kwik-Cast Sealant (World Precision Instruments, Sarasota, FL, USA) to protect it from drying and maintain stable moisture. The stimulation/recording chamber was placed on a stage of an inverted zoom stereo microscope (model SZ02030821 BoliOptics, Rancho Cucamonga, CA, USA).

Bipolar stimulating electrodes were made of two 0.5-mm diameter blunt stainless-steel needles fixed in a custom holder parallel to each other at a fixed distance of 5, 10, or 15 mm. The electrodes were secured vertically in a micromanipulator affixed to the microscope stage. For nerve stimulation, the electrodes were immersed in the solution to a 4-mm depth at the required distance from the nerve. The electrodes’ positions were checked with the microscope and adjusted with the manipulator.

**Figure 8:**
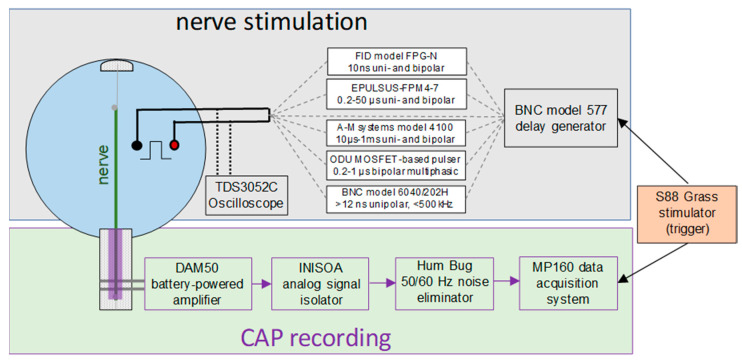
A block diagram of the devices for nerve stimulation (**top**) and recording of the compound action potential (**bottom**). The S88 Grass stimulator on the right triggered both the stimulation and acquisition. BNC model 577 enables the additional control of different pulse generators connected to stimulating electrodes. See text for more details.

The block diagram in Figure 8 explains the connections and triggering of the stimulation and CAP recording equipment. A global trigger for electric pulse generation and data acquisition came from a Grass model S88 stimulator (Grass Instrument Co., Quincy, MA, USA). A model 577 digital delay generator (Berkley Nucleonics, San Rafael, CA, USA) triggered a pulse generator selected for a particular experiment and enabled the additional control of the pulse (phase) duration, burst duration, and pulse repetition rate when needed. We employed six generators with different capabilities to deliver uni- and bipolar stimuli from 10-ns to 1-ms duration and their bursts:

1. The FPG20 generator (FID, Burbach, Germany) produced high-voltage (4–20 kV), 10-ns unipolar or (10 + 10)-ns bipolar pulses with a variable phase amplitude ratio.
2. The EPULSUS-FPM4-7 generator was custom-built by Energy Pulse Systems (Lisbon, Portugal) to deliver positive polarity rectangular pulses from 200 ns to 50 µs at up to 6.5-kV amplitude and 4-MHz rate. It has 4 independently controlled and programmable output channels, and each of the channels can also serve for the current return. Energizing two electrodes in an alternating fashion generated a bipolar electric field between them (see the waveform in Figure 5B). The voltage output of each channel could be tuned either independently or linked to the output of the other channel.
3. The Model 4100 isolated high-power stimulator (A-M systems, Carlsborg, WA, USA) was custom modified for battery-powered operation. It produced nearly rectangular uni- and bipolar stimuli with a phase duration above 1–2 µs and up to 100-V amplitude.
4. A high-voltage, modular, multiphasic nsPEF generator custom-built at ODU [57,58] was employed to generate a triphasic bipolar nsPEF with the first phase of up to 3.2 kV with a sub-microsecond phase duration (see the waveform in Figure 4A).
5. The Model 6040 mainframe with a 202H high-voltage (300 V) plug-in module (Berkley Nucleonics Corporation, San Rafael, CA, USA) produced 15-ns to 5-ms unipolar pulses at up to a 500-kHz repetition rate (see the waveform in Figure 5A).
6. The BNC model 577 digital delay generator could be connected directly to stimulating electrodes to deliver low-voltage (up to 16 V), high-repetition rate (up to 20 MHz) pulses down to 15-ns duration.

The amplitude and shape of the stimulating pulse(s) were continuously monitored with a TDS 3052 oscilloscope (Tektronix, Beaverton, OR, USA).

Elicited CAPs traveled towards the distal end of the nerve and were recorded 8–12 cm away from the stimulation site with a battery-powered DAM50 amplifier (World Precision Instruments, Sarasota, FL, USA). The output of some of the pulse generators employed for stimulation could not be isolated from the ground, so it was imperative to electrically isolate the recording electrodes to prevent possible stimulation effects at the recording site. The amplified signal was passed through an INISOA analog signal isolator (BIOPAC Systems, Goleta, CA, USA) and a Hum Bug noise eliminator (Quest Scientific Instruments, North Vancouver, BC, Canada) to a BIOPAC MP100 Data Acquisition System. With a typical signal noise level of about 10 µV, we recorded the stimulation threshold when the CAP could be readily discerned from the noise at its peak amplitude of about 20 µV (Figure 1A).

### 4.3. Numerical Simulations of the Electric Field Strength

**Figure 9:**
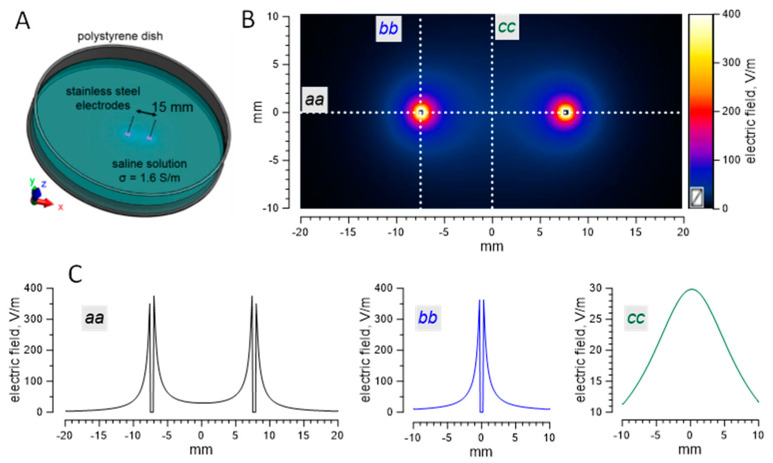
An example of electric field simulations. (**A**) A schematic of the setup used for simulations. (**B**) The electric field distribution in a plane parallel to the bottom of the dish at 2 mm below the saline surface. The values are for 1 V applied between the electrodes (bright spots on the *aa* line). The electric field values along the *aa*, *bb*, and *cc* lines are shown on the graphs in panel (**C**).

Numerical simulations were accomplished with Sim4life Light software (ZMT Zurich MedTech AG. Version 5.2, Zurich, Switzerland). The electrodes were modeled as two stainless-steel cylinders of 0.5-mm diameter spaced 5, 10, or 15 mm apart and placed at 4 mm above the bottom of the Petri dish orthogonal to it. The depth of the solution (1.6 S/m) was 8 mm. The domain of the simulation was discretized to approximately 0.5 million mesh elements 100 µm or 10 µm in size. The electric field was calculated for a horizontal plane 2 mm under the surface of the solution in the absence of the nerve (Figure 9). For a nerve in contact with a stimulation electrode, we used the electric field value calculated for a distance of 100 µm from the electrode surface.

## Data Availability

The data presented in this study are available on request from the corresponding author.

## Abbreviations

CAP	Compound action potential
H-FIRE	High-frequency irreversible electroporation
nsPEF	Nanosecond pulsed electric field
PEF	Pulsed electric field
TMP	Transmembrane potential

## References

[1] Sánchez-Velázquez P., Castellví Q., Villanueva A., Quesada R., Pañella C., Cáceres M., Dorcaratto D., Andaluz A., Moll X., Trujillo M. (2016). Irreversible electroporation of the liver: Is there a safe limit to the ablation volume?. Sci. Rep..

[2] Rubinsky J., Onik G., Mikus P., Rubinsky B. (2008). Optimal Parameters for the Destruction of Prostate Cancer Using Irre-versible Electroporation. J. Urol..

[3] Nuccitelli R., Tran K., Sheikh S., Athos B., Kreis M., Nuccitelli P. (2010). Optimized nanosecond pulsed electric field therapy can cause murine malignant melanomas to self-destruct with a single treatment. Int. J. Cancer.

[4] Al-Sakere B., Andre F., Bernat C., Connault E., Opolon P., Davalos R.V., Rubinsky B., Mir L.M. (2007). Tumor ablation with irreversible electroporation. PLoS ONE.

[5] Tracy C.R., Kabbani W., Cadeddu J.A. (2011). Irreversible electroporation (IRE): A novel method for renal tissue ablation. BJU Int..

[6] Bower M., Sherwood L., Li Y., Martin R. (2011). Irreversible electroporation of the pancreas: Definitive local therapy without systemic effects. J. Surg. Oncol..

[7] Thomson K.R., Cheung W., Ellis S.J., Federman D., Kavnoudias H., Loader-Oliver D., Roberts S., Evans P., Ball C.M., Haydon A. (2011). Investigation of the Safety of Irreversible Electroporation in Humans. J. Vasc. Interv. Radiol..

[8] Scheffer H.J., Nielsen K., de Jong M.C., van Tilborg A.A., Vieveen J.M., Bouwman A.R.A., Meijer S., van Kuijk C., van den Tol P.M., Meijerink M.R. (2014). Irreversible Electroporation for Nonthermal Tumor Ablation in the Clinical Setting: A Systematic Review of Safety and Efficacy. J. Vasc. Interv. Radiol..

[9] Ringel-Scaia V.M., Beitel-White N., Lorenzo M.F., Brock R.M., Huie K.E., Coutermarsh-Ott S., Eden K., McDaniel D.K., Verbridge S.S., Rossmeisl J.H. (2019). High-frequency irreversible electro-poration is an effective tumor ablation strategy that induces immunologic cell death and promotes systemic anti-tumor immunity. Ebiomedicine.

[10] O’Brien T.J., Passeri M., Lorenzo M.F., Sulzer J.K., Lyman W.B., Swet J.H., Vrochides D., Baker E.H., Iannitti D.A., Davalos R.V. (2019). Experimental High-Frequency Irreversible Electroporation Using a Single-Needle Delivery Approach for Nonthermal Pancreatic Ablation In Vivo. J. Vasc. Interv. Radiol..

[11] Beebe S.J., Lassiter B.P., Guo S. (2018). Nanopulse Stimulation (NPS) Induces Tumor Ablation and Immunity in Orthotopic 4T1 Mouse Breast Cancer: A Review. Cancers.

[12] Edelblute C.M., Guo S., Hornef J., Yang E., Jiang C., Schoenbach K., Heller R. (2018). Moderate Heat Application Enhances the Efficacy of Nanosecond Pulse Stimulation for the Treatment of Squamous Cell Carcinoma. Technol. Cancer Res. Treat..

[13] Rossi A., Pakhomova O.N., Pakhomov A.G., Weygandt S., Bulysheva A.A., Murray L.E., Mollica P.A., Muratori C. (2019). Mechanisms and immunogenicity of nsPEF-induced cell death in B16F10 melanoma tumors. Sci. Rep..

[14] Rossi A., Pakhomova O.N., Mollica P.A., Casciola M., Mangalanathan U., Pakhomov A.G., Muratori C. (2019). Nanosecond Pulsed Electric Fields Induce Endoplasmic Reticulum Stress Accompanied by Immunogenic Cell Death in Murine Models of Lymphoma and Colorectal Cancer. Cancers.

[15] Munavalli G.S., Zelickson B.D., Selim M.M., Kilmer S.L., Rohrer T.E., Newman J., Jauregui L., Knape W.A., Ebbers E., Uecker D. (2020). Safety and Efficacy of Nanosecond Pulsed Electric Field Treatment of Sebaceous Gland Hyperplasia. Dermatol. Surg..

[16] Zupanic A., Ribaric S., Miklavčič D. (2007). Increasing the repetition frequency of electric pulse delivery reduces unpleasant sensations that occur in electrochemotherapy. Neoplasma.

[17] Ball C., Thomson K.R., Kavnoudias H. (2010). Irreversible Electroporation: A new chanllenge in “out of operating theater” anesthesia. Anesth. Analg..

[18] Miklavčič D., Pucihar G., Pavlovec M., Ribarič S., Mali M., Maček-Lebar A., Petkovšek M., Nastran J., Kranjc S., Čemažar M. (2005). The effect of high frequency electric pulses on muscle contractions and antitumor efficiency in vivo for a potential use in clinical electrochemotherapy. Bioelectrochemistry.

[19] Teissie J., Golzio M., Rols M.-P. (2005). Mechanisms of cell membrane electropermeabilization: A minireview of our present (lack of?) knowledge. Biochim. Biophys. Acta (BBA)-Gen. Subj..

[20] Gabriel B., Teissie J. (1997). Direct observation in the millisecond time range of fluorescent molecule asymmetrical interaction with the electropermeabilized cell membrane. Biophys. J..

[21] Hibino M., Itoh H., Kinosita K. (1993). Time courses of cell electroporation as revealed by submicrosecond imaging of transmembrane potential. Biophys. J..

[22] Bier M., Hammer S.M., Canaday D.J., Lee R.C. (1999). Kinetics of sealing for transient electropores in isolated mammalian skeletal muscle cells. Bioelectromagnetics.

[23] Pakhomov A.G., Pakhomova O.N. (2020). The interplay of excitation and electroporation in nanosecond pulse stimulation. Bioelectrochemistry.

[24] Azarov J.E., Semenov I., Casciola M., Pakhomov A.G. (2019). Excitation of murine cardiac myocytes by nanosecond pulsed electric field. J. Cardiovasc. Electrophysiol..

[25] Pakhomov A.G., Semenov I., Casciola M., Xiao S. (2017). Neuronal excitation and permeabilization by 200-ns pulsed electric field: An optical membrane potential study with FluoVolt dye. Biochim. Biophys. Acta (BBA)-Biomembr..

[26] Pakhomov A.G., Xiao S., Novickij V., Casciola M., Semenov I., Mangalanathan U., Kim V., Zemlin C., Sozer E., Muratori C. (2019). Excitation and electroporation by MHz bursts of nanosecond stimuli. Biochem. Biophys. Res. Commun..

[27] Semenov I., Grigoryev S., Neuber J.U., Zemlin C.W., Pakhomova O.N., Casciola M., Pakhomov A.G. (2018). Excitation and injury of adult ventricular cardiomyocytes by nano- to millisecond electric shocks. Sci. Rep..

[28] Long G., Shires P.K., Plescia D., Beebe S.J., Kolb J.F., Schoenbach K.H. (2011). Targeted Tissue Ablation With Nanosecond Pulses. IEEE Trans. Biomed. Eng..

[29] Casciola M., Xiao S., Apollonio F., Paffi A., Liberti M., Muratori C., Pakhomov A.G. (2019). Cancellation of nerve excitation by the reversal of nanosecond stimulus polarity and its relevance to the gating time of sodium channels. Cell. Mol. Life Sci..

[30] Gianulis E.C., Casciola M., Zhou C., Yang E., Xiao S., Pakhomov A.G. (2019). Selective distant electrostimulation by synchronized bipolar nanosecond pulses. Sci. Rep..

[31] Pakhomov A.G., Grigoryev S., Semenov I., Casciola M., Jiang C., Xiao S. (2018). The second phase of bipolar, nanosecond-range electric pulses determines the electroporation efficiency. Bioelectrochemistry.

[32] Polajžer T., Dermol–Černe J., Reberšek M., O’Connor R., Miklavčič D. (2020). Cancellation effect is present in high-frequency reversible and irreversible electroporation. Bioelectrochemistry.

[33] Pakhomov A.G., Gudvangen E., Xiao S., Semenov I. (2021). Interference targeting of bipolar nanosecond electric pulses for spatially focused electroporation, electrostimulation, and tissue ablation. Bioelectrochemistry.

[34] Arena C.B., Sano M.B., Rossmeisl J.H., Caldwell J.L., Garcia P.A., Rylander M.N., Davalos R.V. (2011). High-frequency irreversible electroporation (H-FIRE) for non-thermal ablation without muscle contraction. Biomed. Eng. Online.

[35] Mercadal B., Arena C.B., Davalos R.V., Ivorra A. (2017). Avoiding nerve stimulation in irreversible electroporation: A numerical modeling study. Phys. Med. Biol..

[36] Sweeney D.C., Rebersek M., Dermol J., Rems L., Miklavcic D., Davalos R.V. (2016). Quantification of cell membrane permeability induced by monopolar and high-frequency bipolar bursts of electrical pulses. Biochim. Biophys. Acta.

[37] Boinagrov D., Loudin J., Palanker D. (2010). Strength–Duration Relationship for Extracellular Neural Stimulation: Numerical and Analytical Models. J. Neurophysiol..

[38] Bostock H. (1983). The strength-duration relationship for excitation of myelinated nerve: Computed dependence on membrane parameters. J. Physiol..

[39] Reilly J.P. (1998). Applied Bioelectricity: From Electrical Stimulation to Electropathology.

[40] Reilly J.P., Freeman V.T., Larkin W.D. (1985). Sensory effects of transient electrical stimulation-evaluation with a neuroelectric model. IEEE Trans. Biomed. Eng..

[41] Castellví Q., Mercadal B., Moll X., Fondevila D., Andaluz A., Ivorra A. (2017). Avoiding neuromuscular stimulation in liver irreversible electroporation using radiofrequency electric fields. Phys. Med. Biol..

[42] Rogers W.R., Merritt J.H., Comeaux J.A., Kuhnel C.T., Moreland D.F., Teltschik D.G., Lucas J.H., Murphy M.R. (2004). Strength-Duration Curve for an Electrically Excitable Tissue Extended Down to Near 1 Nanosecond. IEEE Trans. Plasma Sci..

[43] Aycock K.N., Zhao Y., Lorenzo M.F., Davalos R.V. (2021). A theoretical argument for extended interpulse delays in therapeutic high-frequency irreversible electroporation treatments. IEEE Trans. Biomed. Eng..

[44] Nesin V., Pakhomov A.G. (2012). Inhibition of voltage-gated Na^+^ current by nanosecond pulsed electric field (nsPEF) is not mediated by Na^+^ influx or Ca^2+^ signaling. Bioelectromagnetics.

[45] Nesin V., Bowman A.M., Xiao S., Pakhomov A.G. (2012). Cell permeabilization and inhibition of voltage-gated Ca^2+^ and Na^+^ channel currents by nanosecond pulsed electric field. Bioelectromagnetics.

[46] Pakhomov A.G., Pakhomova O.N., Pakhomov A.G., Miklavcic D., Markov M.S. (2010). Nanopores: A distinct transmembrane passageway in electroporated cells. Advanced Electroporation Techniques in Biology in Medicine.

[47] Bowman A.M., Nesin O.M., Pakhomova O.N., Pakhomov A.G. (2010). Analysis of plasma membrane integrity by fluorescent detection of Tl^+^ uptake. J. Membr. Biol..

[48] Pakhomov A.G., Bowman A.M., Ibey B.L., Andre F.M., Pakhomova O.N., Schoenbach K.H. (2009). Lipid nanopores can form a stable, ion channel-like conduction pathway in cell membrane. Biochem. Biophys. Res. Commun..

[49] Yang L., Pierce S., Chatterjee I., Craviso G.L., Leblanc N. (2020). Paradoxical effects on voltage-gated Na^+^ conductance in adrenal chromaffin cells by twin vs single high intensity nanosecond electric pulses. PLoS ONE.

[50] Zaklit J., Craviso G.L., Leblanc N., Vernier P.T., Sözer E.B. (2021). 2-ns Electrostimulation of Ca^2+^ Influx into Chromaffin Cells: Rapid Modulation by Field Reversal. Biophys. J..

[51] Gianulis E.C., Lee J., Jiang C., Xiao S., Ibey B.L., Pakhomov A.G. (2015). Electroporation of mammalian cells by nanosecond electric field oscillations and its inhibition by the electric field reversal. Sci. Rep..

[52] Sozer E.B., Pakhomov A.G., Semenov I., Casciola M., Kim V., Vernier P.T., Zemlin C.W. (2021). Analysis of electrostimulation and electroporation by high repetition rate bursts of nanosecond stimuli. Bioelectrochemistry.

[53] Casciola M., Xiao S., Pakhomov A.G. (2017). Damage-free peripheral nerve stimulation by 12-ns pulsed electric field. Sci. Rep..

[54] Brunel N., van Rossum M.C.W. (2007). Quantitative investigations of electrical nerve excitation treated as polarization (Re-printed from *J. Physiol. Pathol. Gen.*
**1907**, *9*, 620–635). Biol. Cybern..

[55] Ibey B.L., Xiao S., Schoenbach K.H., Murphy M.R., Pakhomov A.G. (2009). Plasma membrane permeabilization by 60- and 600-ns electric pulses is determined by the absorbed dose. Bioelectromagnetics.

[56] Gianulis E.C., Casciola M., Xiao S., Pakhomova O.N., Pakhomov A.G. (2018). Electropermeabilization by uni- or bipolar nanosecond electric pulses: The impact of extracellular conductivity. Bioelectrochemistry.

[57] Xiao S., Zhou C., Yang E., Rajulapati S.R. (2018). Nanosecond bipolar pulse generators for bioelectrics. Bioelectrochemistry.

[58] Ryan H.A., Hirakawa S., Yang E., Zhou C., Xiao S. (2018). High-Voltage, Multiphasic, Nanosecond Pulses to Modulate Cellular Responses. IEEE Trans. Biomed. Circuits Syst..

